# Scientifically Formulated Avocado Fruit Juice: Phytochemical Analysis, Assessment of Its Antioxidant Potential and Consumer Perception

**DOI:** 10.3390/molecules26247424

**Published:** 2021-12-07

**Authors:** Arackal Jose Jobil, Sakthivelan Parameshwari, Fohad Mabood Husain, Suliman Yousef Alomar, Naushad Ahmad, Fadwa Albalawi, Pravej Alam

**Affiliations:** 1 Department of Food Technology, Saintgits College of Engineering, Pathamuttom, Kottayam 686532, Kerala, India; scientificreports.nature@gmail.com; 2 Department of Nutrition and Dietetics, Periyar University, Salem 636011, Tamil Nadu, India; 3 Department of Food Science and Nutrition, King Saud University, Riyadh 11451, Saudi Arabia; 4 Department of Zoology, King Saud University, Riyadh 11451, Saudi Arabia; Fadwa_saad@hotmail.com; 5 Department of Chemistry, King Saud University, Riyadh 11451, Saudi Arabia; exactlykot@gmail.com; 6 Department of Biology, College of Science and Humanities, Prince Sattam Bin Abdulaziz University, Alkharj 11942, Saudi Arabia; alamprez@gmail.com

**Keywords:** antioxidant activity, sensory analysis, avocado, optimization, phenolics

## Abstract

The study’s purpose was to find and create a nourishing fruit juice made from avocado to suit nutritional and health demands. In this regard, the avocado juice was formulated using a statistical technique, and its biochemical and phytochemical characteristics were evaluated. Statistically formulated fruit juice was evaluated for its sensory characteristics, proximate composition, nutrients and vitamins, total phenols and flavonoids, and for its antioxidant ability, in addition to a shelf-life test. The optimal amount of all ingredients included in the mathematical model for the preparation of the juice was 150 g of *Persea americana* (Avocado) fruit pulp, 12.5 g of honey and 100 mL of water. In fact, the composition of avocado juice was found to have higher phenolic (910.36 ± 0.215 mg EAG g^−1^/mL) and flavonoid (56.32 ± 1.26 mg QE g^−1^/ mL) amounts. DPPH, ABTS and FRAP antioxidant assays tended to be high compared with a standard. The shelf-life analysis indicated that the processed avocado juice (V7) had a long shelf life. In view of all these merits, a statistically formulated recipe for avocado fruit juice was recommended for the formulation of the most preferred health drink.

## 1. Introduction

A growing knowledge of therapeutic concerns has resulted in greater use of fruit juice and other perishables as a substitutes for conventional beverages such as coffee, tea, or fizzy beverages [1]. Improving food consistency and efficiency has led to breakthroughs in the diversity of fruit juices and beverages [2]. The avocado, of Mexican origin, is known by the name butter fruit. Avocado could be a gateway fruit of the future in India. It possesses a large number of bioactive components and phytochemicals [3] and is responsible for cardiovascular medical advantages. The fruit is reported to play a significant role in defending against the advancement of CHDs (coronary heart diseases) [4,5]. Therapeutic properties with regard to weight, hyperlipidemia, and hyper-cholesterolemia produced by the fruit distinguish it as a functional food [6,7,8]. Avocado is rich in functional lipids such as monounsaturated fatty acids (15.63 g/100 g) and polyunsaturated fatty acids (12.4 g/100 g) [9]. Oleic acid (C18:1), which represents 58.6% of all fatty acids present in butter fruit, is the most common dietary MUFA [10]. Avocado is consumed fresh as a component of salads and spreads, and is also used in guacamole [11]. The smooth consistency of avocado flesh means it is used as a fat replacer in various fruit applications [12]. Consumers desire nutritious food, and researchers have identified that avocado pulp-incorporated juice satisfies consumer demands. Response surface methodology (RSM) is an accumulation of factual and scientific strategies accommodating producing, improving, and upgrading processes [13,14]. The utilization of the RSM in the process enhancement stage means that a test structure is required [15]. From the limitation level, the question of which variables contribute more to the expectation model is resolved, thereby allowing analysts to concentrate on the variables that are most relevant to the response of the object. In the present study, the RSM was utilized to determine the ideal preparation state of avocado juice with a combination of enhancers, as well as its sensory qualities. People are becoming unwell as a result of their present lifestyle and eating habits. This results in a slew of unforeseen health problems. We require a rejuvenating solution to overcome these. With this in mind, we chose the avocado fruit to make a nutritious and nourishing fruit juice. 

## 2. Materials and Methods

### 2.1. Ingredients 

Fully matured and ripened avocado fruits were procured from the Horticulture Research Station (HRS), Kodaikanal, Tamil Nadu. All other ingredients were procured from the local market in Salem district, Tamil Nadu, India.

### 2.2. Juice Preparation and Formulation

The fruit was carefully cleaned with fresh water to eliminate bacteria and dirt. The fruit was then peeled, and the pulp was collected, blended, and used for the experiment [16]. The optimization experiment was performed with honey and water as additives to the formulation.

### 2.3. Optimization Parameters

The formulation of avocado (A), honey (B), and water (C) in the juice was tested and optimized using a centroid mixture method focused on assessing nutritional and sensory characteristics [17,18]. The avocado fruit pulp-incorporated juice recipe and the experimental response variables are given in Table 1 and Table 2. The composition of the juice was enhanced by RSM using a factual plan strategy that used a central composite design to fit a polynomial model via the least-square method (Design-Expertv12, Statease Inc., Minneapolis, MN, USA). For the experimental design, the number of concentrations acquired was dependent on the number of independent variables. 

### 2.4. Experimental Design and Statistics 

Design-Expert software (version.12) was used to develop various responses at the same time. The design of the investigations was intended to create scientific and measurable systems for planning tests and assessing the impacts of variables. The tests we conducted and their reactions were matched to the experimental design. The reactions were broken down after each procedure in order to assess the influence of the free variables on them. The model’s observable criticality was fitted to the responses as follows:

*Y* = *β*_0_ + ∑ *β*_i_
*X*_i_ + ∑ *β*_ii_
*X*_i_^2^ + ∑ *β_ij_ X_i_ X_j_* i                              ii                   ij(1)

where *Y* is the response variable; *β*_0_, *β_i_*, *β*_ii_ and *β*_ij_ are relapsing coefficients; *X*_i_, *X*_j_, and *X*_ij_ are coded free factors.

The response surface methodology (RSM) entails the design of tests, the calculation of factor levels in test runs, the application of suitable scientific models, and, finally, the selection of factor levels by advancing the reaction [13,19]. A central composite rotatable design (CCRD) was utilized to structure the trials, containing three formulating parameters (Table S1). Seventeen runs of investigations were performed, considering three factors *vz.* avocado pulp, honey, and water. 

### 2.5. Sensory Analysis 

The sensory analysis of the formulated fruit juice was performed by following the standard method [20] with some modifications. A total of 150 volunteers were selected to assess the sensory characters of the formulated fruit juice. The volunteers were consumers who preferred fruit juice to soft drinks as they believed in the health benefits of the fruit juices. We gave 150 mL of fruit juice (four servings) to the consumers and asked them to assess the color, mouth-feel and flavor, consistency, and general acceptability. The sensory characteristics were assessed using a nine-point hedonic scale. For each sensory attribute, four responses were gathered from each participant, and the mean values were recorded.

### 2.6. Proximate Composition and Nutrient Analysis

The formulated avocado juice was stored by refrigeration in glass vials for further analysis. Moisture, pH, and total soluble solids (TSS) were analyzed as per the recommended method [21]. Energy, carbohydrate, protein, sugar, and dietary fiber content and titratable acidity were analyzed as per the standard method [22]. Fatty acids were determined by AOCS [23]. The mineral and vitamin contents of the formulated fruit juice were determined following Neilson et al. [24].

### 2.7. Total Phenolic Compounds and Flavonoid Analysis

Total phenolic content was analyzed as per the standard method with some modifications [25,26]. The total phenolics content of the methanolic extract from the formulated avocado fruit juice was determined using the Folin-Ciocalteu method. A gallic acid equivalent curve (GAE) was plotted using gallic acid as the standard. The findings were recorded at 725 nm and reported as mg of gallic acid equivalent (GAE) g^−1^. The flavonoid content of the avocado fruit juice was determined by following the standard method [27]. The methanolic extract was used for determining the flavonoid content and the results were observed at 425 nm with UV–vis spectrophotometry. A quercetin calibration curve was plotted, and the results were expressed as mg quercetin equivalent (EQ) g^−1^ of the avocado sample.

### 2.8. Antioxidant Activity

Following the suggested protocols, an avocado juice methanolic extract was evaluated for in-vitro antioxidant activity [21,28,29]. In brief, 10 mL of methanol (80%) was added to 15 g of the avocado fruit formulation and homogenized. The homogenized suspension was centrifuged at 8000× *g* for 15 min at 4 °C, and the supernatant was used to evaluate the antioxidant activity. 

#### 2.8.1. DPPH assay

A fresh solution of 3.8 mL of 60 μmol/L DPPH was added to different concentrations of methanolic extract (50 to 250 μL) and incubated in the dark for one hour. After incubation, the samples were measured with UV–vis spectrophotometry at 517 nm for absorbance. Ascorbic acid and methanol were used as the control and blank, respectively. The rate of the scavenging activity was determined.

#### 2.8.2. ABTS Assay

ABTS assay for the methanolic extract of the avocado fruit juice was performed as per the standard method [30]. Briefly, 1 mL of the ABTS solution was added to 50 μL of the sample, and the suspension was incubated at room temperature for 30 min. Ascorbic and methanol were used as the control and blank, respectively. After incubation, the solution was assayed at 734 nm with UV–vis spectrophotometry. 

#### 2.8.3. FRAP Assay

In total, 2.5 mL of 1% potassium ferricyanide, prepared in 0.2 M of phosphate buffer, was added to the different concentrations of methanolic extract (50 to 250 μL) and incubated at 50 °C for 20 min. After incubation, 0.5 mL of ferric chloride (0.01%) and 2.5 mL of trichloroacetic acid (10%) were added, mixed well, and incubated for 10 mins. After incubation, the solution was assayed at 700 nm with UV–vis spectrophotometry for ferricyanide iron formation. Ascorbic acid and methanol were used as the control and blank, respectively [28]. 

### 2.9. Shelf-Life Study

The prepared avocado juice was screened for microbial plate counts on a daily basis (from the first to the fifth day) [31].

### 2.10. Statistical Analysis

The deviation between the variables was assessed by a one-way ANOVA and Tukey’s study, and substantial variations were found in the means. Pearson’s correlation of the higher level of confidence (*p* < 0.05 was significant, *p* < 0.001 was highly significant) was evaluated in the relationship between variables. The analysis was streamlined and principal component analysis (PCA) was carried out to determine anomalies. Points between the extremely associated parameters for the individual variables were evaluated utilizing a cluster-embedded heatmap [32].

## 3. Results and Discussion

### 3.1. Avocado Juice Formulation, Optimization, and Sensory Analysis

Avocado is rich in vitamins, plant chemicals, fiber, and minerals, and has a strong antioxidant capability, which is believed to offer protective advantages for consumers against several acute diseases, including diabetes and cardiac diseases [33]. Avocado fruits are believed to confer good health because of their elevated dietary importance, particularly vitamins, antioxidants, fiber, a low sugar level, and a highly valued flavor [34,35]. In this context, we optimized the formulation of avocado fruit juice with the addition of honey as a taste enhancer using RSM to improve the acceptability of the final product. The investigational design involved 17 f tests with actual degrees of various independent factors with scores from 3.25 to 9.74 and the observed reactions to the juice are presented in Table 1. The R2 (coefficient of determination) assessed the fit of the models. 

**Figure 1:**
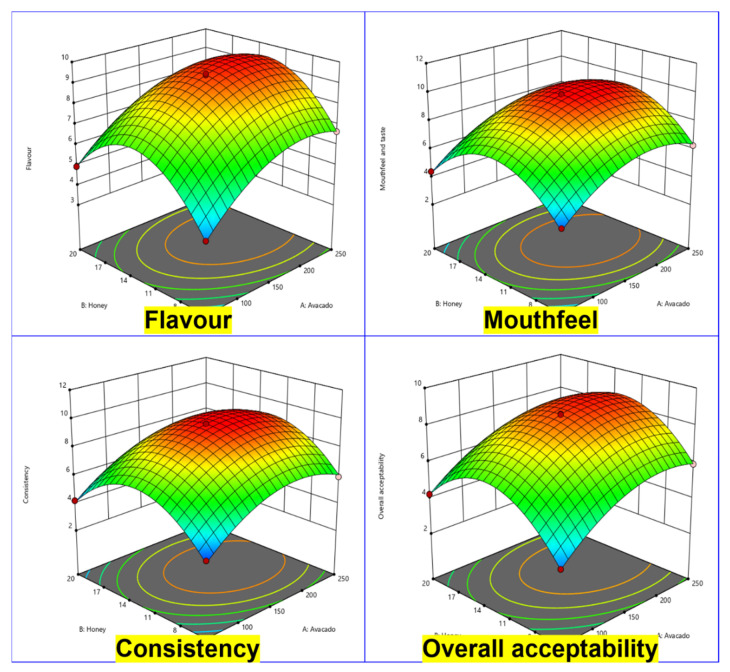
Surface plot represents the flavor attributes of mouthfeel and taste attributes, consistency attributes and overall acceptability attributes of avocado fruit pulp juice formulation.

The regression coefficients, *P* estimates, and model fit statistics of the fitted quadratic models for sensory properties of avocado fruit pulp-incorporated juice helped to assess the sufficiency of each sensory parameter of the developed model. The quadratic models were adequate to clarify the legitimacy of the sensory traits for predicting and exploring purposes inside the structure space [36]. The quadratic condition for predicting the ideal point was acquired by the CCRD structure and info factors; subsequently, the exact connection between the reaction and the free factors in the coded units was exhibited, depending on the trial results. Lucknow and Delahunty [37] shoed that consumers do not compromise on taste for the purpose of health benefits. Hence, the significance of surface model fitting were identified through an ANOVA for flavor, mouth-feel, consistency, and overall acceptability (Figure 1, Table 2 and Table 3). The ANOVA of the quadratic relapse model shows that the model was not critical. This improvement can be ascribed to the blend of at least two sorts of constituents to upgrade the nutrients, minerals, and health benefits of the item [38,39].

The plot ratio was increased based on the sensory and ingredient variations. The estimated optimization was performed for sensory trait parameters such as flavor, mout-feel, consistency, and overall acceptability by making attractive quality limitations [40]. The following equations helped to achieve the desired variation with acceptable sensory characteristics.

Flavor (Y1) = +9.39 + 1.22A + 0.3375B + 1.66C − 0.0900AB + 0.3125AC − 0.0650BC − 1.23A² − 2.44B² − 1.71C² (2)

Mouth-feel and taste (Y2) = +9.87 + 1.09A + 0.0425B + 1.36C − 0.0175AB + 0.4600AC − 0.3425BC − 1.68A² − 2.86B² − 1.53C²(3)

Consistency (Y3) = +9.66 + 1.09A + 0.0800B + 1.30C − 0.0725AB + 0.5400AC − 0.3175BC − 1.75A² − 2.92B² − 1.50C²(4)

Overall acceptability (Y4) = +8.59 + 1.22A + 0.3550B + 1.66C − 0.1000AB + 0.3300AC − 0.0750BC − 1.22A² − 2.40B² − 1.64C²(5)

where A^2^ stands for avocado fruit pulp, B^2^ is honey, and C^2^ is water. 

Table S2 shows the requirements for juice with better tactile properties, alongside the ideal incentives for both free and ward factors. The most extreme score of interest that was accomplished with the ideal estimation of tactile credits was between 3.2 and 9.6. Given these estimations, avocado pulp-based juice was prepared. The observed and predicted qualities were extraordinary (*p* > 0.01), which affirmed the resulting improvement and demonstrated that the anticipated model, V7, was accurate, and the juice can thus be marketed for consumption. 

### 3.2. Proximate Composition and Nutrients of Avocado Juice

**Figure 2:**
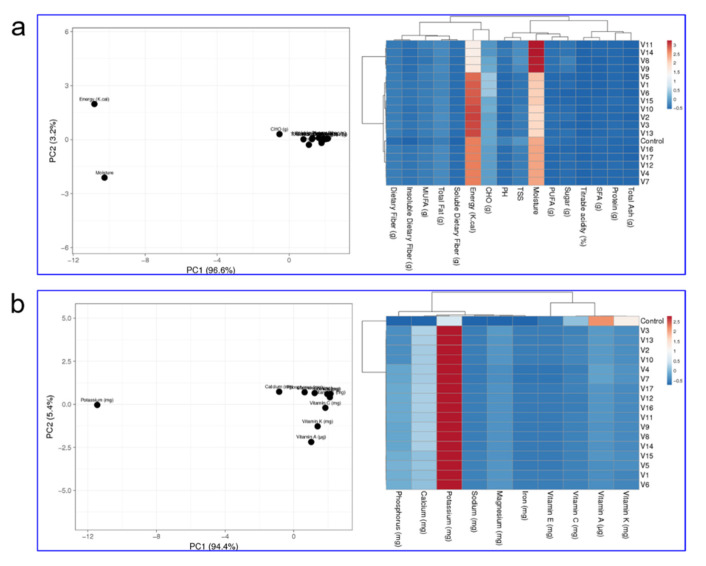
Principal component analysis and cluster-embedded heatmap of sensory characteristics, proximate composition (**a**), vitamin and mineral (**b**) composition of the formulated avocado juice with the aid of statistical optimization.

The proximate composition of the formulated avocado fruit juice is depicted in Figure 2a. The pH values were different among juice samples (2.37 ± 0.23 ^a^ to 6.15 ± 0.05 ^f^). The pH is an acidity or alkalinity indicator of a substance. A pH value of 3 to 4 may give the juice a good likelihood of inhibiting the growth of pathogenic bacteria [40,41]. The TSS of samples varied significantly and the values ranged from 8.59 ± 0.13 ^a^ to 14.74 ± 0.32 ^e^ mg/L. V6 had higher (14.74 ± 0.32 ^e^ mg/L) TSS than the control sample (12.88 ± 2.10 ^bc^ mg/L). The higher TSS in V6 is s attributed to the presence of a higher proportion of suspended solids. Titratable acidity is a measure of the acid present in the juice. The titratable acidity of the control was higher (1.25 ± 0.16 ^e^%) than the variants. The titratable acidity of the variations ranged from 0.12 ± 0.01 to 0.88 ± 0.10 percent. 

The energy content of avocado juice ranged from 49.06 ± 0.75 ^a^ to 313.00 ± 1.03 kcal/g. The energy content of the avocado juice standard was 89 ± 0.19 kcal/g. When the avocado juice substitution was increased, there was a significant increase in energy content. Avocado is a medium energy-dense (1.7 kcal/g) fruit because it contains about 80% water and dietary fibers [35]. The carbohydrate content of avocado juice ranged from 10.60 ± 0.20 ^a^ to 88.13 ± 2.19 g/mL. Avocado’s mesocarp is rich in sugars, amino acids, carbohydrates, and many other metabolites that show high variations in terms of concentration [42,43]. The edible part (pulp) is fleshy and contains 65 to 80% water; 1 to 4% protein; 1 to 2% sugar and 3 to 30% oil. The carbohydrate content of the standard was 16.51 ± 0.75 g/mL. The total fat content was 6.1 ± 0.01 g/mL for the standard. The variations ranged from 7.43 ± 0.11 ^a^ to 30.53 ± 0.05 g/mL. Avocado contains about 14% fat. This directly affected the fat level of the juice.

The protein content of avocado juice combinations ranged from 1.36 ± 0.32 ^a^ to 6.13 ± 0.05 ^i^ g/mL. The protein content of the standard was 1.11 ± 1.61 g/mL. This showed that the substitution of avocado juice up to 250 mL increased the protein content of the juice. Moisture content is an important parameter that contributes to the palatability of juice. The moisture content of the variations was higher than the standard (79.82 ± 0.25%). The moisture content of the variations ranged from 84.50 ± 0.51a to 89 ± 0.58%. The ash content is an indication of the mineral content of the prepared samples. The ash content of the juice ranged from 0.2433 ± 0.00 ^a^ to 2.06 ± 0.05 ^h^ g/mL, while the ash content of the standard was 0.31 ± 0.99 g/mL. The saturated fatty acid content of avocado juice ranged from 0.4767 ± 0.06 to 2.86 ± 0.03 g/mL and the standard value was 1.61 ± 0.05 g/mL. The monounsaturated fatty content of the standard was 2.41 ± 0.41 g/mL and the variation ranged from 4.11 ± 0.00 ^a^ g to 23.06 ± 0.37 ^f^ g/mL. The polyunsaturated fatty acid content of the variations ranged from 2.24 ± 0.00 to 5.48 ± 0.02 g/mL and the standard was 2.51 ± 0.12 g/mL. The sugar content of the standard was 1.61 ± 0.21 g/mL. The sugar content of the avocado juice variations ranged from 1.47 ± 0.06 ^a^ to 6.48 ± 0.06 g/mL. 

The dietary fiber content of the prepared avocado juice was higher than the standard (1.44 ± 0.74 g/mL). The dietary fiber content of avocado juice contains a high level of fiber, ranging from 4.51 ± 0.06 ^a^ to 22.15 ± 0.56 g/mL. Epidemiological data suggest that high intake of dietary fiber reduces the risk of coronary heart disease [44]. The fiber content, which included both soluble and insoluble forms, could contribute toward cholesterol reduction. The soluble dietary fiber content of the standard was 0.12 ± 0.01 g/mL, and in the prepared samples ranged from 2.10 ± 0.00 ^a^ to 8.24 ± 0.17 g/mL. The insoluble dietary fiber of the prepared avocado juice ranged from 2.17 ± 0.00 ^a^ to 14.18 ± 0.11 ^j^ g/mL, and the values were higher than the standard commercial avocado sample (0.01 ± 0.13 g). 

The mineral and vitamin contents of the formulated avocado juice are represented in Figure 2b. The calcium content of juice prepared with avocado ranged from 64.11 ± 0.00 to 265.15 ± 0.02 mg/mL against the standard of 0.15 ± 0.00 mg/mL. This confirmed that the calcium content of the prepared sample was greater than that of the standard juice. The potassium content of the prepared juice ranged from 280.11 ± 0.01 to 1400.13 ± 0.057 mg/mL, and the standard was 45.17 + 0.13 mg/mL. The potassium contents of the prepared avocado juice variations were higher than those of the standard. The magnesium content of the standard was 0.109 + 0.09 mg/mL, and the values of the prepared samples ranged from 19.36 ± 0.06 to 97.34 ± 0.15 mg/mL. The phosphorus content of the juice ranged from 35.14 ± 0.01 to 191.64 ± 0.16 mg/mL. The phosphorus content of the standard was 191.83 ± 0.01 mg/mL. The phosphorus content increased with the increased addition of avocado pulp. The sodium and iron content ranged from 4.58 ± 0.06 ^b^ to 29.31 ± 0.71 mg/mL and 1.19 ± 0.12 to 4.26 ± 0.12 mg/mL, and their standards were 28.26 ± 0.15 and 4.26 ± 0.16 mg/mL, respectively.

The vitamin A content of the standard was 103.63 ± 1.33 µg/mL. V5 obtained the highest vitamin A content (103.46 ± 0.05 µg) due to the increased addition of avocado pulp in the juice. V9 obtained a low vitamin A value (20.38 ± 0.14 µg/mL) because of the low amount of avocado in the juice. The vitamin K content of the standard was 70.12 ± 0.00 µg. V15 obtained a high vitamin K content (70.43 ± 0.01 µg/mL) compared to the other variations, again because of the increased levels of avocado pulp in the juice. 

V11 has a low vitamin K value (14.40 ± 0.16 µg/mL) because of the low amount of avocado in the juice. Ascorbic acid is a water-soluble compound that is essential for life. Fruit juices need to contain it since the body cannot produce it by itself. Ascorbic acid was found to be present in the prepared avocado juices, although in slightly lower quantities (6.25 ± 0.23–29.69 ± 0.17 mg/mL) compared to the commercial juices (30.26 ± 0.20 mg/mL). The higher concentration of vitamin C in commercial juice is due to its addition during production (ingredients of commercial juice: avocado, water, ascorbic acid, E-142 artificial coloring agents, benzoic acid, citric acid, methyl eugenol). The highest vitamin C content was present in the V15 variation (29.69 ± 0.17 mg/mL) with the presence of a higher quantity of avocado pulp. The vitamin E content of V5 (7.77 ± 0.29 mg/mL) was the highest compared to the standard (7.55 ± 0.11 ^cd^ mg/mL).

### 3.3. Phenolics and Flavonoids

The results of the phytochemical analysis are given in Table S3. The various biological activities recorded that are relevant to avocado residue extractions are larvicidal, antifungal, antimicrobial, antiprotozoal, antidiabetic, antihypertensive, hypocholesterolemic, antimycobacterial, and those that inhibit fat or protein oxidation [45,46]. Rangel-Sánchez et al. [47] identified reactive oxygen species and antioxidant responses to injuries in unripe avocado fruit. Rodriguez-Sanchez et al. [48] documented the isolation and chemical recognition of lipid derivatives with immunomodulatory and antithrombotic activity. The free radical scavenging actions were presumably induced by flavonoids and tannins because these are phenolic products that are strong antioxidants and free radical scavengers [49,50].

The key components for antioxidant ability are phenolic compounds in plants [51,52]. This analysis found that total phenolic content ranged from 180.29 ± 0.145 to 910.36 ± 0.215 mg EAG g^−1^/mL. V1 exhibited the highest level of phenolic compounds, with 910.36 ± 0.215 mg EAG g^−1^/mL. V8, V9, and V11 showed lower values of phenolic compounds, possibly due to the very small percentage of avocado in the juice. The Hass variety had 12.6, 4.9 and 51.5 mg EAG g^−1^/mL dry sample amounts for peel, pulp and seed in a report by Wang et al. [53], which assessed the antioxidant capacities and phenolic compounds of eight varieties of avocado. These findings are lower than in the current pulp analysis. This may be due to the different solvents and conditions of extraction. Flavonoids are antioxidants, i.e., oxidative cell scavengers that have a powerful anti-carcinogenic effect and safeguard the tissue in all phases of tumorigenesis. In terms of flavonoid content, V1 stood out, with 56.32 ± 1.26 mg QE g^−1^/mL, demonstrating stronger outcomes than any of the others. 

### 3.4. Antioxidant Activity

#### 3.4.1. DPPH Assay

**Figure 3:**
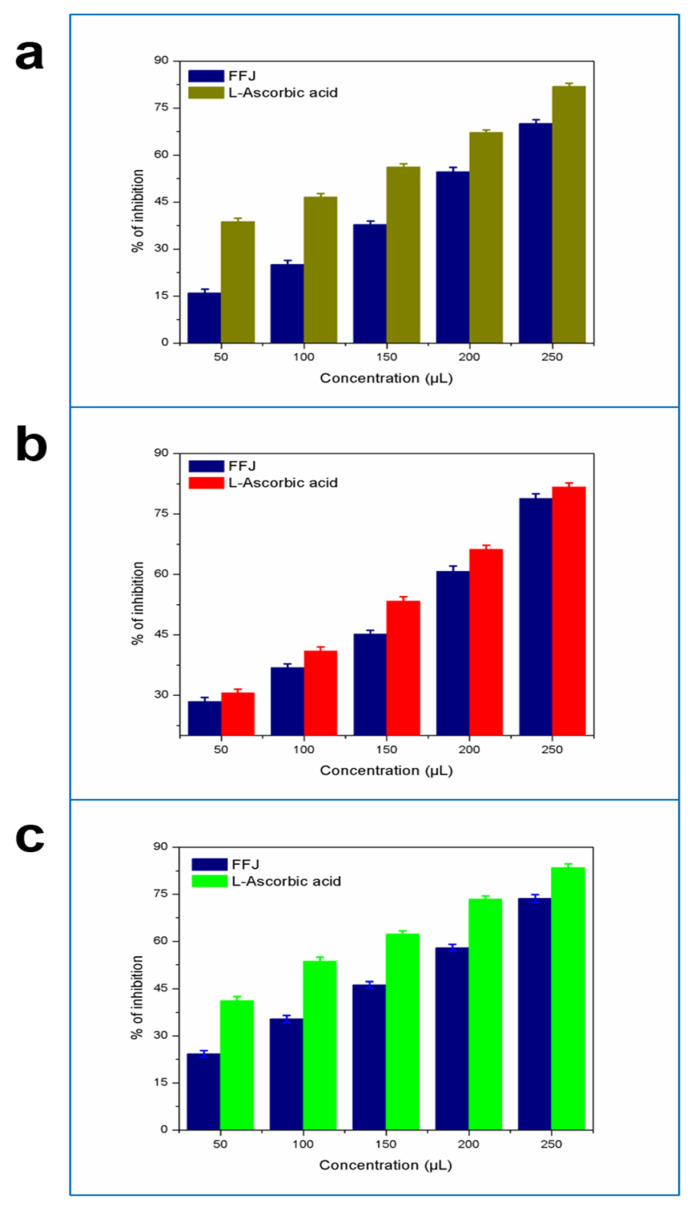
Antioxidant activities evaluated for the formulated avocado fruit juice using the DPPH (**a**)**,** ABTS (**b**)**,** and FRAP (**c**) assays.

The DPPH radical scavenging method was used to test the antioxidant role of formulated fruit juice and establish the requirement of 50% inhibitors of radicals in the methanol extract concentration. Previous research found that avocado mesocarp juice had greater antioxidant activity than avocado seeds [54,55]. Hydro-ethanol extracts of peels had higher total phenolic content (227.9 mg/g) compared to seeds, while seeds had a lower antioxidant capacity (EC50 = 220 μg/mL) compared to peels (EC50 = 149 μg/mL) in the DPPH scavenging assay [56]. Additionally, according to the DPPH assay in our study, which measures the ability of antioxidants to scavenge the DPPH radical, higher antioxidant activity was noted in 250 µL avocado fruit juice (V7), presenting 78.92% of the inhibition of antioxidant activity observed when compared to the control, with 81.76% of the inhibition of activity (Figure 3a). The fruit extract containing phenolics and flavonoids, which are secondary metabolite compounds that are abundant in fruit extracts, had very high antioxidant activity.

#### 3.4.2. ABTS Assay

The outcome of antioxidant activity by the ABTS test relies on the production of a blue and green ABTS test utilizing dissolved substances in organic compounds [57]. The discrepancy in the antioxidant potential calculated by the dual assays seemed to be larger in high-quality foods such as mesocarp and peas [58]. The ABTS free radical scavenging activity rankings ranged from 269.56 ± 6.52 μmol TE g^−1^/mL to 442.72 ± 9.62 μmol TE g^−1^/mL. Similarly, our results showed a high antioxidant activity in the ABTS assay at 250 µL, presenting 70.81 μmol TE g^−1^/mL of the inhibition of the antioxidant activity observed when compared to the control in ascorbic acid, with 81.92 mg/g of the inhibition of the activity of avocado fruit extract (Figure 3b). This potentially makes avocado fruit extract a better source of antioxidative agents.

#### 3.4.3. FRAP Assay

The reduction of Fe^3+^ to Fe^2+^ by antioxidant substances is noticeable when the yellow color of the test solution changes to different shades of green, while development of blue color is found in Ferric reducing power tests. Our study results showed a higher antioxidant activity in the 250 µL methanolic extract with 70.81 μmol Fe (II)/mL of inhibition compared to the control ascorbic acid (81.92 mg/g). The 50 µL methanolic extract exhibited a reduced power activity of 24.31 μmol Fe (II)/mL, which was less than the control at 41.02 μmol Fe (II)/mL (Figure 3c). Finally, we claim that the enhanced activity of antioxidants is fairly attributed to flavonoid and phenolic substances, thereby responding to the antioxidant activity capacity shown by the FRAP study. Rotta et al. [59] reported a comparison of avocado-peel teas to other commercially manufactured teas, showing that the tea is close to that of avocado peel, which is mostly consumed for its antioxidant properties [60]. The analysis of antioxidant function by the FRAP did not vary significantly during storage.

### 3.5. Shelf Life 

Shelf life is an essential property of every form of food and is of concern to all in the food chain, from manufacturer to customer. The product may suffer changes in flavor, odor, composition, or appearance at the end of the shelf life that are deemed unacceptable or unwanted. Boadu [60] states that chemical reactions, primarily through oxidation or Maillard reactions, microbial reactions, and biochemical reactions triggered through natural enzyme-facilitated interactions, lead to a loss of consistency, such as enzyme browning, and may be the underlying cause of the shift. Variations in texture can often be due to physical responses, while the root cause may be biochemical [61].

The formulated juices were examined initially and every alternate day up to the fifth day (Table S4) for their microbial content in order to evaluate the shelf life of the juice. The total microbial load of the avocado juice ranged from 7 × 10^−6^ to 31 × 10^−6^ CFU/g. On the first day, the level in the standard and variations was at its minimum. The counts increased throughout the storage period. A variety of experiments were performed to validate the antibacterial efficacy of extracts of avocado [62]. However, the growth of microorganisms until the fifth day was within the permissible limit as per BIS standards (IS 11536:2006). Therefore, it is recommended that the product can be consumed up to the fifth day of storage. 

## 4. Conclusions

This research focused on the experimental composition of avocado fruit juice. The prepared fruit juice was evaluated for its sensory characteristics, proximate composition, nutrients and vitamins, total phenols and flavonoids, and its antioxidant ability, as well as its shelf life. The optimal amount of all ingredients included in the mathematical model for the preparation of juice was 150 g of avocado fruit pulp, 12.5 g of honey and 100 mL of water. The composition of avocado juice was found to have higher phenolic and flavonoid amounts, which were substantially impacted by their antioxidant activity. In comparison, the DPPH, ABTS and FRAP-based assays showed higher antioxidant activity compared to the standard. A shelf-life analysis indicated that the processed avocado juice had a long shelf life. Given all these merits, a statistically formulated recipe for avocado fruit juice was recommended for the formulation of the most preferred health drink.

## Figures and Tables

**Table 1:** Central composite face-centered design with experimental values of response variables.

Run	Independent Variable (Ingredients)			Dependent Variable (Sensory Attributes)			
	Avocado Pulp (g) (A)	Honey (mL) (B)	Water (mL) (C)	Flavor	Mouthfeel and Taste	Consistency	Overall Acceptability
1	250	20	100	7.14 ± 0.20 ^e^	6.28 ± 1.11 ^ab^	5.91 ± 0.35 ^cd^	6.42 ± 4.42 ^cd^
2	150	20	150	7.11 ± 0.87 ^e^	6.59 ± 0.98 ^ab^	6.38 ± 0.28 ^c^	6.24 ± 0.62 ^c^
3	150	5	50	3.25 ± 0.74 ^a^	3.68 ± 0.50 ^a^	3.47 ± 0.31 ^a^	2.54 ± 0.57 ^a^
4	150	12.5	100	9.34 ± 0.73 ^f^	9.87 ± 0.82 ^b^	9.66 ± 0.12 ^e^	8.59 ± 0.89 ^de^
5	250	5	100	6.67 ± 0.38 ^de^	6.30 ± 3.01 ^ab^	5.93 ± 0.17 ^c^	5.91 ± 0.34 ^c^
6	250	12.5	50	5.65 ± 0.48 ^cd^	6.29 ± 2.75 ^ab^	5.88 ± 0.57 ^c^	4.92 ± 0.41 ^bc^
7	150	12.5	100	9.41 ± 0.82 ^f^	9.87 ± 0.01 ^b^	9.65 ± 0.21 ^e^	8.56 ± 0.09 ^de^
8	50	20	100	4.94 ± 0.50 ^bc^	4.41 ± 2.35 ^a^	4.20 ± 0.55 ^ab^	4.23 ± 0.24 ^abc^
9	50	12.5	150	6.61 ± 0.55	6.32 ± 1.89 ^ab^	5.86 ± 0.75 ^c^	5.89 ± 0.32 ^c^
10	150	5	150	6.54 ± 0.89 ^de^	7.13 ± 2.76 ^ab^	6.82 ± 0.33 ^d^	5.86 ± 0.75 ^c^
11	50	12.5	50	3.77 ± 1.16 ^ab^	4.56 ± 2.55 ^a^	4.45 ± 0.95 ^b^	3.05 ± 0.34 ^ab^
12	150	12.5	100	9.34 ± 1.26 ^f^	9.87 ± 0.17 ^b^	9.60 ± 0.17 ^e^	8.54 ± 0.95 ^de^
13	150	20	50	4.07 ± 0.78 ^ab^	4.50 ± 3.01 ^a^	4.30 ± 0.56 ^ab^	3.39 ± 1.09 ^ab^
14	50	5	100	4.11 ± 0.86 ^ab^	4.32 ± 3.02 ^a^	3.93 ± 0.58 ^ab^	3.32 ± 0.19 ^ab^
15	250	12.5	150	9.74 ± 0.73 ^f^	9.69 ± 0.51 ^b^	9.45 ± 0.58 ^e^	9.04 ± 0.17 ^e^
16	150	12.5	100	9.39 ± 0.20 ^f^	9.58 ± 0.52 ^b^	9.65 ± 0.21 ^e^	8.62 ±0.28 ^de^
17	150	12.5	100	9.45 ± 0.53 ^f^	9.58 ± 0.52 ^b^	9.59 ± 0.15 ^e^	8.71 ± 0.33 ^de^

Significant differences among the variables are based on row.

**Table 2:** Results of regression analysis of avocado fruit pulp incorporated juice (flavor, mouth feel and taste).

Factors	Flavor				Mouth-Feel and Taste			
	SS^2^	DF	F	*p*-Value	SS^2^	DF	F	*p*-Value
Model	83.6	9	1057.81	0.0001	87.66	9	427.94	<0.0001
A	11.9	1	1358.18	0.0001	9.48	1	416.64	<0.0001
B	0.91	1	103.73	0.0001	0.0145	1	0.6349	0.4517
C	22.01	1	2505.59	0.0001	14.82	1	651.30	<0.0001
AB	0.0324	1	3.69	0.0963	0.0012	1	0.0538	0.8232
AC	0.3906	1	44.46	0.0003	0.8464	1	37.19	0.0005
BC	0.0169	1	1.92	0.2080	0.4692	1	20.62	0.0027
A^2^	6.41	1	730.13	0.0001	11.81	1	519.02	<0.0001
B^2^	25.00	1	2845.88	0.0001	34.50	1	1515.80	<0.0001
C^2^	12.30	1	1400.25	0.0001	9.86	1	433.05	<0.0001
Residual	0.0615	7			0.1593	7		
Lack of fit	0.0526	3	7.86	0.0375	0.1593	3		

SS^2^ = Sum of squares; DF = degree of freedom; F = f values; A = avocado fruit pulp; B = honey, C = water.

**Table 3:** Results of regression analysis of avocado fruit pulp incorporated juice (consistency and overall acceptability).

Factors	Consistency				Overall Acceptability			
	SS^2^	DF	F	*p*-Value	SS^2^	DF	F	*p*-Value
Model	88.96	9	285.60	0.0001	81.61	9	1286.9	<0.0001
A	9.53	1	275.25	0.0001	11.91	1	1689.9	<0.0001
B	0.0512	1	1.48	0.2633	1.01	1	143.09	<0.0001
C	13.55	1	391.38	0.0001	22.04	1	3128.8	<0.0001
AB	0.0210	1	0.6075	0.4613	0.0400	1	5.68	0.0487
AC	1.17	1	33.70	0.0007	0.4356	1	61.82	0.0001
BC	0.4032	1	11.65	0.0112	0.0225	1	3.19	0.1171
A^2^	12.89	1	372.56	0.0001	6.22	1	882.92	<0.0001
B^2^	35.84	1	1035.49	0.0001	24.26	1	3443.62	<0.0001
C^2^	9.47	1	273.72	0.0001	11.26	1	1598.5	<0.0001
Residual	0.2423	7			0.0493	7		
Lack of fit	0.2423	3			0.0442	3	11.51	0.0195

SS^2^ = Sum of squares; DF = degree of freedom; F = f values; A = avocado fruit pulp; B = honey, C = water.

## Data Availability

Not applicable.

## Supplementary Materials

The following are available online. Table S1: Coded categorical variables with three categories, Table S2: Predicted optimization of process parameters by desirability approach, Table S3: Total phenolics and flavonoids content of the formulated avocado fruit juice, Table S4: Microbial count of formulated avocado juice recorded during the shelf life study.
