# Supplementary material for: Scientifically Formulated Avocado Fruit Juice: Phytochemical Analysis, Assessment of Its Antioxidant Potential and Consumer Perception
Source: Molecules. 2021 Dec 7;26(24):7424. doi: 10.3390/molecules26247424 (PMC8706416; doi:10.3390/molecules26247424)
Supplement: Supplementary file 1 [file molecules-26-07424-s001.zip › molecules-1467506-supplementary.pdf]

Supplementary material

Consumer Perception of Scientifically Formulated Avocado Fruit Juice and Assessment of its Antioxidant Potential and Essential Phytochemicals

Arackal Jose Jobil[1,2], Sakthivelan Parameshwari[2*], Fohad Mabood Husain[3], Suliman Y Alomar[4], Naushad Ahmad[5], Fadwa Albalawi[6] and Pravej Alam[7]

Table S1. Coded categorical variables with three categories

**Independent variables**	**Coded value**		
	**-1**	**0**	**+1**
Avocado pulp (A)	50	150	250
Honey (B)	5	12.5	20
Water (C)	50	100	150

Table S2. Predicted optimization of process parameters by desirability approach

**Process Parameters**	**Target**	**Experimental Design**		**Optimum values**	**Importance**
Avocado fruit pulp	Maximize	50	250	150	3
Honey	Range	5	`	12.5	3
Water	Range	50	150	100	3
**Responses**					
Flavour	Range	3.2	9.7	5.4	3
Mouthfeel	Range	3.6	9.8	6.5	3
Consistency	Range	3.4	9.6	5.4	3
Overall acceptability	Range	2.5	9.4	6.8	3

Table S3. Total phenolics and flavonoids content of the formulated avocado fruit juice.

Variables	Total phenolic (mg EAG g-1)	Flavonoids (mg EQ g-1)
V1	910.36	56.32
V2	546.69	34.46
V3	546.49	31.37
V4	544.13	35.15
V5	860.37	55.68
V6	904.27	54.32
V7	544.13	35.15
V8	184.34	11.59
V9	181.56	11.56
V10	538.03	34.84
V11	180.29	10.81
V12	544.13	35.15
V13	546.13	35.24
V14	186.01	10.69
V15	905.83	57.5
V16	544.13	35.15
V17	544.13	35.15

Table S4. Microbial count of formulated avocado juice recorded during the shelf life study.

Variables	No. of colonies			CFU/ml			Dilution
	Day 1	Day 3	Day 5	Day 1	Day 3	Day 5	
V1	7	11	19	7×10^{-6}	11×10^{-6}	19×10^{-6}	10^{-6}
V2	5	12	18	5×10^{-6}	12×10^{-6}	18×10^{-6}	10^{-6}
V3	11	18	27	11×10^{-6}	18×10^{-6}	27×10^{-6}	10^{-6}
V4	12	17	28	12×10^{-6}	17×10^{-6}	28×10^{-6}	10^{-6}
V5	9	13	26	9×10^{-6}	13×10^{-6}	26×10^{-6}	10^{-6}
V6	16	22	31	16×10^{-6}	22×10^{-6}	31×10^{-6}	10^{-6}
V7	14	19	25	14×10^{-6}	19×10^{-6}	25×10^{-6}	10^{-6}
V8	12	18	29	12×10^{-6}	18×10^{-6}	29×10^{-6}	10^{-6}
V9	14	21	33	14×10^{-6}	21×10^{-6}	33×10^{-6}	10^{-6}
V10	15	20	34	15×10^{-6}	20×10^{-6}	34×10^{-6}	10^{-6}
V11	14	18	28	14×10^{-6}	18×10^{-6}	28×10^{-6}	10^{-6}
V12	12	19	29	12×10^{-6}	19×10^{-6}	29×10^{-6}	10^{-6}
V13	13	21	31	13×10^{-6}	21×10^{-6}	31×10^{-6}	10^{-6}
V14	13	24	33	13×10^{-6}	24×10^{-6}	33×10^{-6}	10^{-6}
V15	13	21	32	13×10^{-6}	21×10^{-6}	32×10^{-6}	10^{-6}
V16	12	18	27	12×10^{-6}	18×10^{-6}	27×10^{-6}	10^{-6}
V17	14	20	31	14×10^{-6}	20×10^{-6}	31×10^{-6}	10^{-6}
